# Regular-fat and low-fat dairy foods and cardiovascular diseases: perspectives for future dietary recommendations

**DOI:** 10.1016/j.ajcnut.2025.03.009

**Published:** 2025-03-13

**Authors:** Benoît Lamarche, Arne Astrup, Robert H Eckel, Emma Feeney, Ian Givens, Ronald M Krauss, Philippe Legrand, Renata Micha, Marie-Caroline Michalski, Sabita Soedamah-Muthu, Qi Sun, Frans J Kok

**Affiliations:** 1 Centre Nutrition, santé et société (NUTRISS), Institut sur la nutrition et les aliments fonctionnels, Université Laval, Quebec City, QC, Canada; 2 Department of Obesity and Nutrition Sciences, Novo Nordisk Foundation, Hellerup, Denmark; 3 University of Colorado School of Medicine, Aurora, CO, United States; 4 Institute of Food and Health, University College Dublin, Dublin, Ireland; 5 Institute for Food, Nutrition & Health, University of Reading, Reading, United Kingdom; 6 Departments of Pediatrics and Medicine, University of California, San Francisco, CA, United States; 7 Laboratoire de Biochimie-Nutrition Humaine, Institut Agro/INSERM NuMeCan, Rennes, France; 8 Department of Food Science and Nutrition, University of Thessaly, Greece; 9 Friedman School of Nutrition Science and Policy, Tufts University, Boston, MA, United States; 10 Universite Claude Bernard Lyon1, CarMeN Laboratory, INSERM, INRAE, 69310, Pierre-Bénite, France; 11 Department of Medical and Clinical Psychology, Center of Research on Psychological disorders and Somatic diseases, Tilburg University, Tilburg, The Netherlands; 12 Departments of Nutrition and Epidemiology, Harvard T.H. Chan School of Public Health, Boston, MA, United States; 13 Channing Division of Network Medicine, Department of Medicine, Brigham and Women’s Hospital and Harvard Medical School, Boston, MA, United States; 14 Division of Human Nutrition and Health, Wageningen University, Wageningen, The Netherlands

**Keywords:** cardiovascular disease (CVD), dietary guidelines, low-fat dairy, regular-fat dairy, saturated fatty acids (SFAs)

## Abstract

Most current dietary guidelines for the prevention of cardiovascular diseases (CVD) recommend the consumption of low-fat dairy in place of regular-fat dairy foods, one of the main sources of dietary saturated fatty acids (SFAs). Here, we summarize the data presented and discussions held—relating to the validity of such recommendations—between a panel of international nutrition research experts at a high-level closed workshop on "Saturated Fat in Dairy and Cardiovascular Diseases," which took place in Amsterdam on 15–16 April, 2024. The most recent evidence indicates that overall, consumption of milk, yogurt and cheese, irrespective of fat content, is neutrally associated with CVD risk. There is also no evidence yet from randomized controlled trials that consumption of regular-fat milk, yogurt, and cheese has different effects on a broad array of cardiometabolic risk factors when compared with consumption of low-fat milk, yogurt, and cheese. Thus, the body of evidence does not support differentiation between regular-fat and low-fat dairy foods in dietary guidelines for both adults and children. Strategies focusing primarily on reduction of energy-dense, nutrient-poor foods, the main source of SFAs in Western diets, rather than on the fat content of dairy foods, are more likely to benefit the population’s cardiovascular health. Future research is needed to understand better the place of regular-fat and low-fat dairy foods within healthy eating patterns.

## Introduction

Most dietary guidelines around the world recommend minimizing the consumption of SFAs to prevent cardiovascular disease (CVD) risk [[1], [2], [3]], often with a target that SFAs should represent <10% of total energy intake (<10%E) [[4], [5], [6]]. Such recommendations are, in part, based on relatively undisputed evidence from experimental human studies that substituting unsaturated fats for SFAs reduces LDL cholesterol [1,2,7,8], a key etiological risk factor for CVD [[7], [8], [9], [10], [11]]. Yet, the recommendation to limit SFA as a key dietary target for CVD prevention has recently been challenged [12,13], with some experts arguing that there is no definite scientific rationale to support the <10%E cutoff for SFA intake [[14], [15], [16]]. Dietary SFAs come from a wide range of animal- and plant-based products, the major sources of SFAs in many Western diets being industrially processed foods, followed by meat and dairy products. For example, data in adults from the Canadian Community Health Survey 2015 indicated that 67.8% of all SFAs were from meat products and foods not recommended in Canada’s Food Guide 2019 whereas dairy foods contributed to <25% of total SFA intake [17]. In the United States, data from the 2017–2020 National Health and Nutrition Examination Survey of participants aged 2+ y are similar, with 41.6% of total SFA intake being from foods other than dairy, meat, seafood, and plant-based products; dairy contributed to 28.4% of all SFA intake [18]. Compelling evidence suggests that the food matrix influences the physiological and health effects of nutrients, including SFAs [19,20]. For example, both the emulsified state of fat and the structure of lipid molecules influence the digestibility and metabolism of dietary SFAs [21,22]. Additional evidence from prospective cohort studies using substitution modeling shows that the replacement of meat with dairy products as a source of SFAs is associated with a lower risk of CVD, including incident coronary artery disease (CAD) [[23], [24], [25], [26]]. This emerging evidence reflects the inconsistent association between SFAs and CVD risk and has implications for the relevance of recommendations in most dietary guidelines to consume low-fat, in place of regular-fat, dairy foods.

Consequently, a meeting of international nutrition sciences experts was held in April 2024 to share state-of-the-art research and expert insights on the current state of scientific knowledge regarding the association between dairy foods with various fat contents and CVD. The aim was to reach a consensus on whether there is sufficient evidence to position regular-fat and low-fat dairy foods differentially in dietary recommendations. This paper summarizes the discussions held at that meeting, including a brief review of the current scientific knowledge from relevant epidemiological, clinical, and mechanistic studies, provides insights on the position of regular-fat and low-fat dairy foods in dietary guidelines, and identifies knowledge gaps to be filled by research to support future guidance.

The definition of regular-fat and low-fat dairy foods varies across countries and studies. For this work, low-fat refers to dairy foods that are considered as being reduced in fat as per most guidelines, for example, <2% fat milk or 3% fat yogurt. Low-fat cheese refers to cheeses that typically contain <6% fat or are 25% lower in fat than the concentration of their referenced food. The term ‘regularfat’ used in this paper encompasses terms such as ‘full-fat’ or ‘whole fat’.

## What is the Epidemiological Evidence Associating Dairy Food Consumption and Dairy Fat with CVD Risk?

**FIGURE 1:**
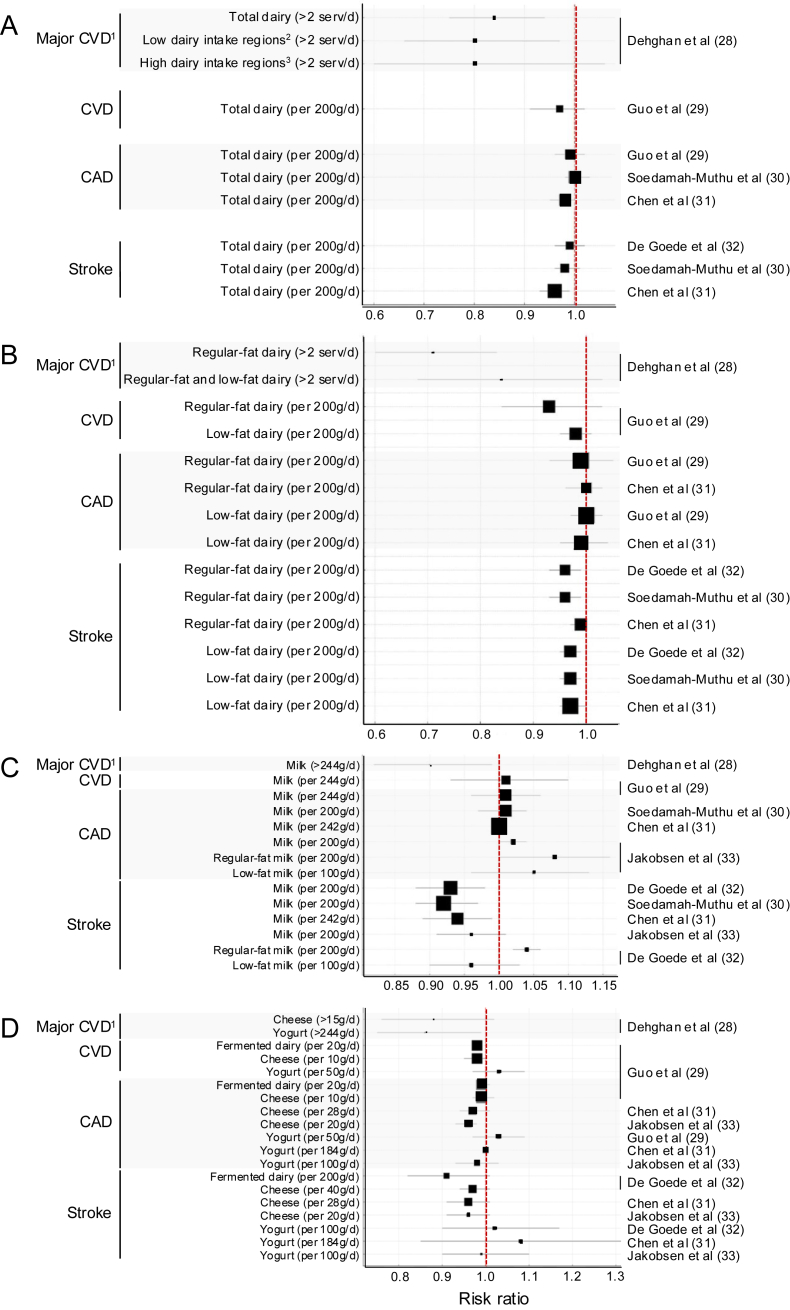
Dose–response results of large prospective cohort studies and meta-analyses examining the association of dairy food consumption and risk of cardiovascular outcomes; (A) total dairy, (B) regular-fat and low-fat dairy, (C) milk, (D) fermented dairy, cheese and yogurt. Square sizes are proportional to study sample size within each panel. Dehghan et al. [28] reported results from the Prospective Urban Rural Epidemiology cohort study of nearly 140,000 adults from 21 countries in 5 continents over a median follow-up of 9 y. ^1^Major cardiovascular disease (CVD) was defined as death from cardiovascular causes, nonfatal myocardial infarction, stroke, or heart failure; ^2^low dairy intake regions included China, south Asia, southeast Asia, Africa; ^3^high dairy intake regions included Europe and North America, South America, and the Middle East. Guo et al. [29] reported results from a meta-analysis of 29 prospective cohort studies in over 900,000 adults from North America, Europe, Australia, and East Asia over a mean follow-up of 13 y. Fermented dairy foods included cheese, yogurt, and soured milk. Soedamah-Muthu et al. [30] reported updated results from Guo et al. 2017, including 15 cohort studies for coronary artery disease (CAD), and De Goede et al. 2016, including 20 cohort studies for stroke. Chen et al. [31] reported results from a meta-analysis of 55 studies in over 850,000 adults from North America, Europe, and Asia over a follow-up time of 5–32 y. De Goede et al. [32] reported results from a meta-analysis of 18 prospective cohort studies in over 750,000 adults from North America, Europe, Australia, and East Asia over a follow-up of 8–26 y. Jakobsen et al. [33] reported results from a meta-analysis of 18 prospective cohort studies in over 600,000 adults from Europe, North America, and Asia over a follow-up time of 5–23 y. Yogurt was defined as yogurt or other soured milk products. Across studies, regular-fat and low-fat dairy categories usually included milk, yogurt, and cheese. Low-fat milk/dairy was usually defined as having fat content lower than regular-fat milk/dairy, although for cheese, this could include both reduced-fat cheeses and naturally low-in-fat cheeses.

A systematic review of meta-analyses published before 2016 indicated that consumption of various forms of dairy products in observational studies, including regular-fat and low-fat dairy foods, showed either favorable or neutral associations with CVD-related clinical outcomes [27]. The quality of evidence supporting these associations (or lack thereof) ranged from moderate to high. There was no evidence that consumption of any individual types of dairy food, including milk, cheese, and yogurt, was associated with a higher risk of CVD. Since this 2016 systematic review, several meta-analyses including more recent prospective cohort studies on the association between dairy consumption and CVD risk have been published. Results from these meta-analyses, along with the large, multinational Prospective Urban Rural Epidemiology (PURE) observational cohort study are summarized in Figure 1 [[28], [29], [30], [31], [32], [33]]. Risk estimates in these studies are adjusted for a large spectrum of diet-related confounding factors. Butter, cream, and plant-based dairy alternatives were not included as they are not considered as dairy foods in most dietary guidelines.

**FIGURE 2:**
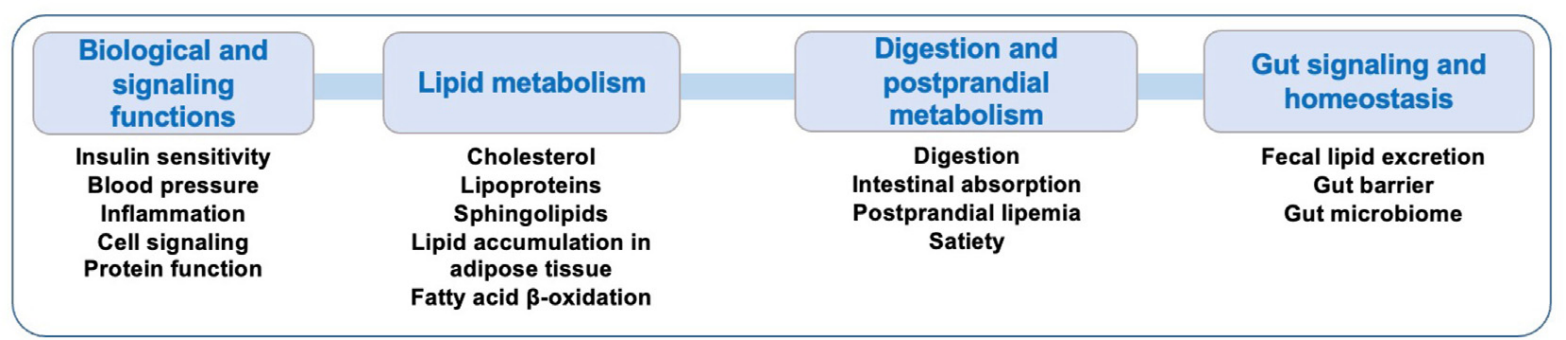
Potential mechanisms linking dairy food consumption and dairy fat to cardiometabolic health. Several mechanisms from cellular and animal models, as well as from clinical studies in humans, have associated components of dairy foods to beneficial cardiometabolic changes [[61], [62], [63], [64]]. Potential benefits on biological and signaling functions include contribution to acylation processes that are essential to some protein/enzyme functions (notably those involved in omega-3 metabolism), the impact of several dairy-specific fatty acids (such as trans-vaccenic acid and trans-palmitoleic acid) on insulin sensitivity, hypotensive effects of dairy biopeptides and anti-inflammatory effects of dairy fats. Some dairy SFAs consist of short- and medium-chain fatty acids that are readily β-oxidized and have been associated with various potentially beneficial effects on lipid metabolism. The consumption of milk polar lipids and milk fat globule membrane have been shown to reduce LDL cholesterol, apolipoprotein B, and triacylglycerol concentrations, to reduce intestinal cholesterol absorption (human/randomized controlled trial) and cholesterol liver storage, increase lipid β-oxidation, and reduce lipid accumulation in tissues (preclinical) [22]. Milk polar lipids may also reduce circulating concentrations of atherogenic sphingolipids (such as ceramide Cer24:1) [65]. Specific milk fatty acids may also reduce lipid accumulation in the liver and adipose tissue. Other potentially beneficial effects of dairy components on digestion and postprandial lipid metabolism have been documented. For example, the structure and texture of the dairy matrix may modulate digestion, gastric emptying rate, and postprandial lipemia [66]. Consumption of yogurt and cheese has been shown to favorably influence satiety, notably via glucagon-like peptide 1 mediated signaling. The calcium and polar lipid content of the dairy matrix, and its rigid texture, may reduce intestinal absorption of some fatty acids, cholesterol, and/or polar lipids. Finally, dairy components may influence gut signaling and homeostasis*,* including favorable modulation of the gut microbiome and the production of beneficial postbiotics such as short-chain fatty acids.

**FIGURE 3:**
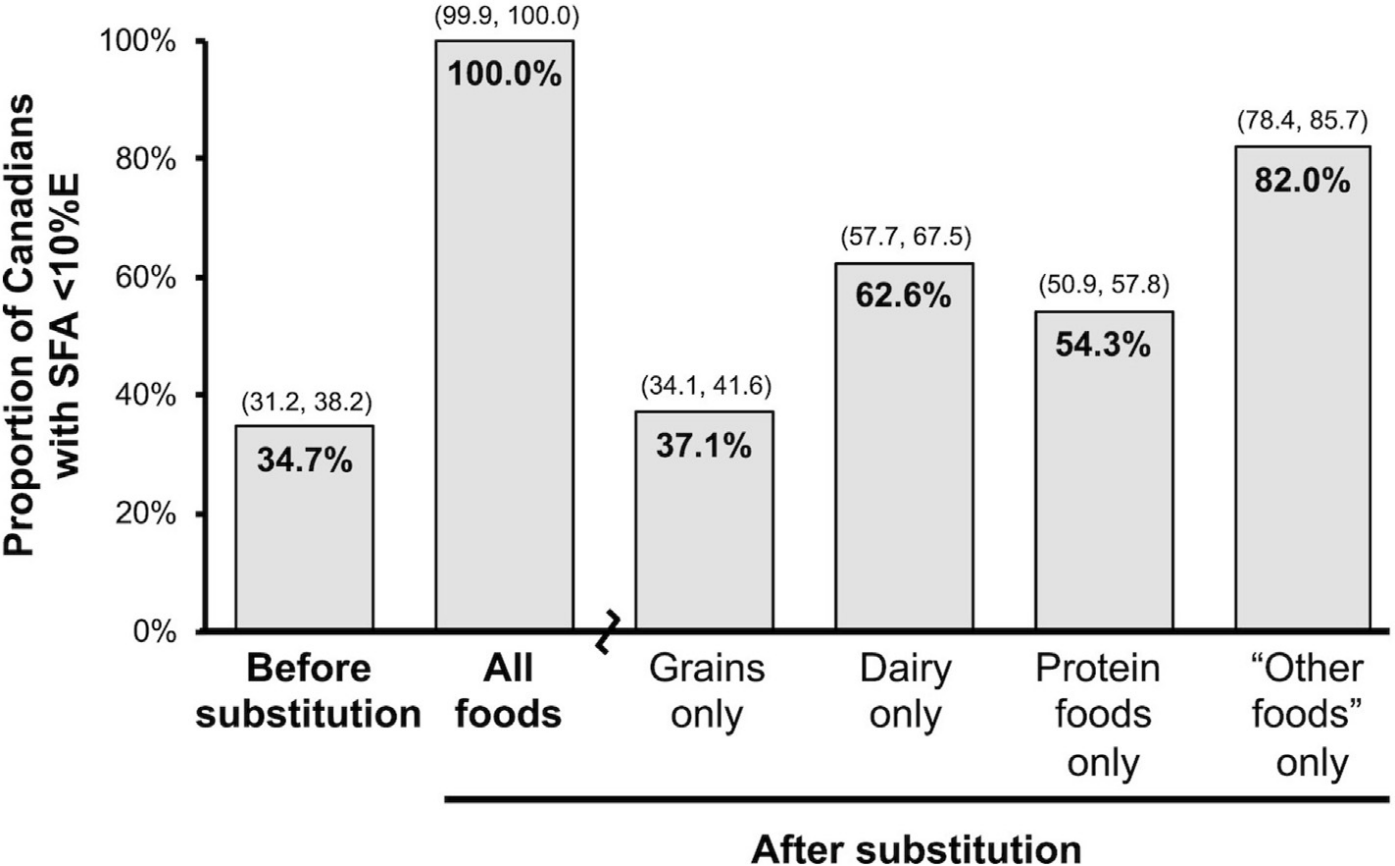
Effects of food substitution on the proportion of Canadians in 2015 with saturated fatty acid intake <10% of total energy. Modeling of the proportion of the Canadian population (aged 2 y or more) with usual total SFA intakes <10% of total energy before and after the substitution of foods high in SFA with the corresponding foods that are low in SFA/high in UFA. Reproduced with permission from Harrison et al. [73]. 10%E, 10% of total energy intake; UFA, unsaturated fatty acid.

Key findings from the PURE study and these recent meta-analyses are the following: *1*) total dairy intake is either not associated with or favorably associated with risk of CVD, including CAD and stroke (Figure 1A); *2*) there is no conclusive evidence that consumption of regular-fat or low-fat dairy foods is differentially associated with any CVD-related clinical outcomes (Figure 1B), with the exception of regular-fat milk, which may be associated with a higher risk of CAD or stroke (Figure 1C); *3*) consumption of fermented dairy, primarily yogurt and cheese, in general shows no association with risk of CVD (Figure 1D). Results from these studies align with other observations that dairy food consumption is not associated with overall mortality risk or with incident type 2 diabetes, a condition that increases risk of CVD [[28], [29], [30],^34^].

## Discussion of Findings from Observational Epidemiology

Meta-analyses of randomized controlled trials (RCTs) and of cohort studies are generally regarded as robust and high-quality evidence to inform dietary guidelines. Data from the most recent meta-analyses of prospective cohort studies confirm previous observations that dairy food consumption in general is either favorably associated or not associated with CVD risk [[27], [28], [29], [30], [31], [32], [33],^35^]. In one meta-analysis, higher intakes of regular-fat milk were associated with a slight increase in risk of stroke, whereas higher intakes of low-fat milk were not [32]. However, the small number of studies included in this meta-analysis (*N* = 4) and the fact that 1 cohort study was responsible for most of the small effect size observed indicates that more studies are needed to confirm these findings. In another meta-analysis, higher intakes of regular-fat milk were associated with a small but significantly higher risk of CAD, although the authors highlighted the substantial heterogeneity across the small number of studies analyzed (*N* = 6) [33]. Nevertheless, these findings are at odds with reported favorable or null associations and potential effects of milk consumption on risk of hypertension, atherosclerosis, stroke, and major CVD events [28,[36], [37], [38]]. A greater consumption of cheese, which is much higher in fat and SFA than regular-fat milk, was associated with a lower risk of CAD in the same meta-analysis. In that context, it was generally agreed at the meeting that the body of evidence remains too limited to support a differentiation in dietary guidelines between regular-fat and low-fat dairy with regard to CVD risk prevention. The possibility that intakes of regular-fat and low-fat milk are differentially associated with risks of stroke and CAD therefore needs to be substantiated with more data.

Although essential to informing nutrition-focused public health recommendations, observational studies are limited by their capacity to account for food substitution. This is a key consideration because risks associated with the consumption of a given food largely depend on the food it is replacing within a food pattern. In epidemiological studies using statistical modeling approaches to food or nutrient substitution, isoenergetic substitution of SFAs with unsaturated fats has been associated with a lower CVD risk, with PUFAs demonstrating the greatest benefits, followed by MUFAs and whole-grain carbohydrates [[39], [40], [41]]. However, this nutrient-based substitution approach does not account for the fact that food sources of SFAs may mitigate some of their purported health effects [26,34]. Substitution modeling using observational data suggests that replacing dairy foods with red meat, especially processed meat, is associated with higher CVD risk [23,24,26,42,43], whereas replacement of dairy foods with plant-based foods, such as nuts, legumes, and whole grains, may be associated with more favorable cardiometabolic health outcomes [25,39,44]. Although these models are potentially informative, we propose that more comprehensive substitution modeling should be undertaken in cohort studies, including dose–response studies, to advance our understanding of the overall health implications of substituting regular-fat or low-fat dairy for other foods within a variety of complex dietary patterns. Such studies should also include analyses of the impact of food substitutions on the intake of key nutrients, beyond just SFA.

Most evidence from prospective studies is from European and North American cohorts characterized by moderate consumption of dairy foods, with an upper range of ∼3 servings per day. The PURE study found consistent cardiovascular benefits associated with relatively higher dairy consumption in regions characterized as having either low or high dairy intakes [28]. However, a meta-analysis has shown stronger favorable associations between milk intake and risk of stroke in East Asian populations with a low dairy intake (median intake 38 g/d) than in Western populations with a high dairy intake (median intake 266 g/d) [32]. More studies are needed in regions of both low and high dairy intake and where serving size and composition of dairy foods are different from the typical consumption in Europe and North America, to establish potential dose–response relationships between regular-fat and low-fat dairy intake and CVD risk.

Finally, the relevance of combining all dairy foods into a single category in epidemiological studies is not recommended because milk, yogurt, and cheese, irrespective of fat content, are very different foods. Grouping dairy foods solely based on fat content is also not recommended. Indeed, the fat content of a low-fat cheese, which may vary from 5% to 25%, is much higher than the fat content of a low-fat milk, which typically ranges from 0% to 1% [30,32]. Regular-fat and low-fat dairy foods may also be heterogeneous in terms of sugar content and additives. Epidemiological research on the fat content of dairy foods is also limited by the quality and accuracy of databases on these foods, especially given that definitions of regular-fat and low-fat dairy foods vary substantially across studies and jurisdictions.

In summary, most guidelines around the world propose that dairy foods can be part of a healthy dietary pattern. Many guidelines also recommend that low-fat dairy should be favored over regular-fat dairy. We suggest that there is currently insufficient evidence from epidemiology to support this ubiquitous recommendation to consume low-fat dairy foods in place of regular-fat dairy foods for CVD prevention.

## What is the Evidence from RCTs Regarding the Effects of Dairy Food Consumption and Dairy Fat on Cardiometabolic Risk?

No RCT has been carried out to assess the long-term effects (in terms of years) of foods such as dairy on clinical CVD outcomes because of the numerous challenges associated with such an undertaking. However, rigorously analyzed RCTs can evaluate the short-term effects (in terms of weeks or months) of food intake on cardiometabolic risk factors associated with CVD risk such as blood lipids and lipoproteins, blood pressure, body weight and composition, glycemic control and inflammation. The impact of dairy food consumption on CVD-related cardiometabolic risk factors has been summarized in several recent meta-analyses of RCTs (Table 1) [45,46]. Although butter is not considered a dairy food in most dietary guidelines, comparisons with butter highlight how the matrix of different sources of SFA, including dairy fat sources, may differentially influence LDL cholesterol concentrations and other cardiometabolic risk factors. This is important because dairy foods are one of many dietary sources of SFA at the population level with a common assertion that higher consumption of regular-fat dairy foods leads to increases in blood total cholesterol and LDL cholesterol concentrations.

**TABLE 1:** Results from meta-analyses of randomized controlled trials examining the impact of dairy food consumption on cardiometabolic risk factors.

Cardiometabolic risk factor	Study and food source comparison, mean difference (95% CI)			
	Kiesswetter et al. [45] High dairy vs. control/low overall dairy^1^	Kiesswetter et al. [45] High regular-fat dairy vs. control/low overall dairy^1^	Kiesswetter et al. [45] High low-fat dairy vs. control/low overall dairy^1^	Pradeilles et al. [46] Cheese vs. butter^2^
Blood lipids				
Total cholesterol (mmol/L)	N/A	N/A	N/A	–0.24 (–0.34, –0.15)
LDL cholesterol (mmol/L)	0.08 (–0.06, 0.23)	0.24 (–0.21, 0.68)	0.00 (–0.26, 0.27)	–0.19 (–0.27, –0.12)
HDL cholesterol (mmol/L)	0.03 (–0.02, 0.09)	0.26 (0.03, 0.49)	0.05 (–0.06, 0.16)	–0.04 (–0.08, –0.00)
Triglycerides (mmol/L)	–0.03 (–0.12, 0.07)	–0.08 (–0.52, 0.37)	–0.07 (–0.29, 0.15)	0.03 (–0.01, 0.07)
Blood pressure				
Systolic BP (mm Hg)	–1.37 (–4.31, 1.56)	–7.60 (–17.18, 1.97)	–5.22 (–10.01, –0.43)	N/A
Body weight and composition				
Body weight (kg)	–0.17 (–0.92, 0.59)	–3.15(–12.43, 6.13)	0.23 (–0.82, 1.28)	N/A
Body mass index (kg/m^2^)	0.10 (–0.34, 0.54)	N/A	–0.01 (–0.46, 0.43)	N/A
Fat mass (kg)	–0.59 (–1.74, 0.56)	0.31 (–8.30, 8.93)	1.02 (–1.04, 3.09)	N/A
Waist circumference (cm)	–1.29 (–3.29, 0.70)	2.76 (–6.01, 11.53)	–1.99 (–5.96, 1.98)	N/A
Glycemic control				
Fasting glucose (mmol/L)	0.03 (–0.14, 0.20)	0.43 (0.00, 0.86)	0.31 (0.03, 0.60)	N/A
Glycated hemoglobin (%)	0.07 (–0.08, 0.21)	0.37 (0.12, 0.61)	0.47 (0.15, 0.78)	N/A

Abbreviations: BP, blood pressure. All values are mean differences with 95% confidence intervals in parentheses; N/A, not available.

1 Kiesswetter et al. [45] conducted a network meta-analysis of 19 randomized controlled trials with 1427 participants, which had a ≥12-wk intervention comparing high overall, regular-fat, or low-fat dairy intake (≥3 servings per day or equal amount in grams per day; regular-fat or low-fat definitions based on the common fat contents of dairy products) and low dairy/control intake (0–2 servings per day or usual diet irrespective of fat content).

2 Pradeilles et al. [46] conducted a pooled analysis of 7 randomized controlled trials including 264 participants investigating the effect of isoenergetic substitution of 135 g/d hard or semihard cheese intake with ∼52 g/d butter intake on blood lipid markers after ≥14 d.

As shown in Table 1, results from meta-analyses of RCTs on regular-fat and low-fat dairy food consumption do not support that belief. Moreover, several RCTs have demonstrated that consumption of hard or semihard cheese lowers total cholesterol and LDL cholesterol concentrations compared with consumption of butter, despite contributing to similar PUFA/SFA ratios [[46], [47], [48]]. Lower concentrations of total cholesterol and LDL cholesterol have also been observed with consumption of dairy foods including regular-fat dairy foods enriched with probiotics compared with those without probiotics [49]. Several meta-analyses of RCTs have also shown that the consumption of both regular-fat or low-fat dairy foods had no effect on HDL cholesterol or triglyceride levels compared with low amounts or no dairy [45,50,51]. However, evidence supporting any differential effects of regular-fat and low-fat dairy on blood lipids remains limited because of the small number of studies in which both were directly compared. More studies are needed to resolve this question.

Evidence also suggests no effect of increasing dairy food consumption on most nonlipid cardiometabolic risk factors including blood pressure, body weight and composition, glycemic control, and inflammation [45,51,[52], [53], [54]]. This seems to be the case irrespective of fat content of the dairy foods [45,51]. An intervention based on the Dietary Approaches to Stop Hypertension diet found comparable reductions in blood pressure when regular-fat and low-fat dairy foods were included in the experimental diets [1]. Similarly, higher intakes of dairy foods compared with low amounts or no dairy, irrespective of fat content, had no detrimental effects on body weight and composition, although effects may vary according to experimental conditions related to energy restriction [45,52]. The effect of dairy consumption on glucose homeostasis remains uncertain, with evidence from RCTs suggesting marginal detrimental effects [45,53]. The long-term impacts of such small changes in glycemic control are unclear, because dairy intake has not been associated with a higher risk of type 2 diabetes in epidemiological studies [30,34]. However, consumption of probiotic yogurt has been associated with significant reductions in levels of fasting glucose and glycated hemoglobin compared with nonprobiotic yogurt [55]. The Food and Drug Administration recently determined that there is credible evidence supporting a relationship between higher yogurt intake and lower risk of type 2 diabetes, irrespective of fat content [56]. Finally, data from one meta-analysis of RCTs suggested that increased dairy intake may have marginal anti-inflammatory effects [54], although as indicated previously, this has not been a systematic finding.

## Discussion of Findings from RCTs

The RCTs available provide relatively consistent evidence that the consumption of various forms of dairy foods has marginal effects on cardiometabolic risk, including on raising LDL cholesterol concentrations. However, the results of these studies should be interpreted with several considerations in mind. In general, many dietary intervention studies are conducted in young, healthy male Caucasian volunteers, who are not representative of the wider population. Most RCTs were of short duration and not designed *a priori* to examine multiple outcomes, with the possibility that many were underpowered to detect changes in cardiometabolic risk factors that were measured as secondary or exploratory outcomes.

As indicated previously, the recommendation to consume low-fat in place of regular-fat dairy foods is largely based on the purported LDL cholesterol raising effect of dietary SFA, to which dairy foods contribute [1,2,7]. This recommendation does not appear to be supported by evidence from RCTs. Furthermore, previous studies have found that dairy SFAs increase levels of large, buoyant LDL particles rather than levels of small, dense LDL particles [[57], [58], [59]]. However, the extent to which these findings might influence the impact of regular-fat compared with low-fat dairy foods on CVD risks remains uncertain [60]. Together, observations from RCTs that consumption of regular-fat and low-fat dairy are not differentially associated with surrogate markers of CVD risk are relatively consistent with data from observational studies of CVD outcomes.

## Evidence that the Dairy Food Matrix Influences Biological Mechanisms

Several factors in the composition of dairy foods interact to modulate cardiometabolic risk. These include nutrient content and fatty acid composition, fat molecular structure (for example, triglyceride fatty acid profile and polar lipids within the milk fat globule membrane) and supramolecular structure (for example, fat droplet size), matrix composition and structure (influenced by food processing and fermentation), and the make-up of bioactive peptides released upon consumption [[61], [62], [63], [64]]. The potential impacts of these interactions are depicted in Figure 2 [22, [61], [62], [63], [64], [65], [66]]. Briefly, dairy fat and the dairy food matrix resulting from the processing of dairy food production have been shown to have several biological and signaling functions, to influence lipid and cholesterol metabolism, to alter digestion and the postprandial lipid response, as well as to modify gut signaling and gut homeostasis [22,[65], [66], [67]]. Studying this interplay of acting and counteracting biological mechanisms may provide insights into potential mechanisms that ultimately result in the relatively neutral effects that dairy food consumption has on cardiometabolic risk. We argue that categorizing dairy foods primarily based on their SFA content does not do justice to the diversity of these foods. We acknowledge that most evidence of this interplay emerges from studies in animal and in vitro models, with limited yet rapidly growing evidence in humans through RCTs. More research is therefore warranted to confirm in humans many of the purported mechanisms through which dairy foods and dairy fat modulate cardiometabolic and CVD risk.

## Key Observations and Recommendations

Epidemiological and clinical research on dairy foods currently does not provide evidence differentiating the associations between regular-fat and low-fat dairy products with CVD risk or differentiating their effects on cardiometabolic risk in adults. The topic of children was not discussed specifically at the workshop that led to this paper. However, the small number of studies available to date in this population also highlights the lack of evidence differentiating the effects of regular-fat and low-fat dairy foods on cardiometabolic risk factors among children [68]. There is even evidence from observational studies suggesting that consumption of regular-fat milk compared with low-fat milk in children is associated with lower odds of overweight or obesity [69]. Current recommendations promoting the consumption of low-fat in place of regular-fat dairy foods to limit SFA intake are therefore not evidence-based. Such recommendations, which are ubiquitous in dietary guidelines around the world, may in fact be a distraction among other healthy eating recommendations, for which the evidence is much more convincing.

It is increasingly recognized that overall dietary patterns have a greater relevance to cardiovascular health than SFA intake alone [13,70]. Broad recommendations to reduce SFA intake may also result in a lower intake of key nutrients such as vitamin D, calcium, iodine, and vitamin B_12_ if implemented without appropriate replacement strategies [2,71], whereas food-based dietary guidance can ensure lowering SFA intake although helping to maintain intake of these key nutrients [12,72]. For example, dairy foods in the United States make up ∼28% of dietary sources of SFAs, with meat accounting for another 25%, plant-based foods <10% and other food sources—including industrially processed foods—over 40% [18]. A study among Canadians has shown that in 2015, 35% of the population achieved the recommended <10%E cutoff for SFA intake [73]. Substitution modeling studies in this population have shown that this proportion would increase to 63% if all dairy foods consumed by the population were low-fat dairy. However, similar simulations also showed that the replacement of nondairy foods high in SFA with corresponding low-SFA, high unsaturated fatty acid replacement foods within the "other foods" category of Canada’s Food Guide 2019 accounted for the greatest predicted reduction in usual SFA intakes among Canadians, with 82% of the population achieving the <10%E target for SFA intake (Figure 3) [73]. Reducing intake of SFAs from "other" foods such as unprocessed and processed meats, salty snacks or pastries, rather than from dairy foods, is likely to be a more effective way to reduce SFA intake as well as reducing consumption of energy-dense, nutrient-poor foods such as ready-to-eat meals. Low-fat dairy foods often contain additives such as texturizers, which have recently raised health concerns [74,75]. In addition, the excess SFAs generated by removing the fat from regular-fat dairy products to create low-fat dairy products may re-enter the food chain through nutrient-poor food categories such as snacks or ready-to-eat meals. This raises the question as to whether dietary fat should remain within its natural foods. Finally, although the participants at the workshop did not specifically discuss climate-change-related issues, more research on the impact of the evolving dairy industry is needed to identify optimal dairy intake in light of the health of human beings and the planet.

In conclusion, differentiating low-fat from regular-fat dairy in dietary recommendations is currently not supported by the available evidence in adults. We propose that:

1) dietary guidelines for adults should emphasize food-based strategies that are likely to have a greater impact on a population’s SFA intake, by focusing primarily on the replacement of energy-dense, nutrient-poor foods, the main source of SFAs at the population level;
2) healthy dietary patterns recommended in North America and in Europe are generally high in minimally processed plant-based foods and in whole grains and low in red or processed meats and in highly processed snacks or meals. Such patterns, independent of intake of dairy fat, will inevitably lead to important reductions in SFA intake at the population level;
3) further research is needed to understand better the potential associations between dietary patterns that include various forms of dairy foods with varying amounts of fat and CVD risk in a broad variety of populations with different risk profiles.

## Author contributions

BL: primary responsibility for final content; and all authors: attended the closed workshop on "Saturated Fat in Dairy and Cardiovascular Diseases" and contributed to workshop discussions and to the subsequent design, data collection and interpretation, writing and review of this manuscript, read and approved the final manuscript.

## Funding

The authors reported that no funding was received for this study.

## Conflict of interest

The Dutch Dairy Association had no role in the discussions held at the high-level closed workshop and did not participate or provide comments during the development and writing of this manuscript. AA is a member of the Journal’s Editorial Board and is also an Associate Editor on *The American Journal of Clinical Nutrition* and played no role in the journal’s evaluation of the manuscript, reports a relationship with Rééducation Nutritionnelle et Psycho-Comportementale Scientific Committee and *American Journal of Clinical Nutrition* that includes board membership; and a relationship with Ferrero that includes funding grants. QS reports travel provided by Dutch Dairy Association. AA, RHE, IG, EF, RMK, PL, RM, M-CM, SS-M, and FJK reports financial support and travel provided by Dutch Dairy Association. BL reports writing assistance provided by Chill Pill Media Ltd and relationship with Health Canada that includes funding grants. EF reports a relationship with Food for Heath Ireland and Teagasc Food Research Ireland that includes funding grants; relationship with Irish section of the Nutrition Society and *British Journal of Nutrition* that includes board membership; relationship with National Dairy Council Ireland that includes consulting or advisory and travel reimbursement. IG reports a relationship with Global Dairy Platform, Dairy Australia, Barham Benevolent Foundation, UK Research and Innovation, Medical Research Council that includes funding grants; relationship with European Milk Federation, French National Interprofessional Centre for Dairy Economics, and Dairy Council Northern Ireland that includes speaking and lecture fees and travel reimbursement; relationship with ELSEVIER INC that includes consulting or advisory. RMK reports a relationship with Dairy Management Inc that includes funding grants. RM reports a relationship with National Institutes of Health and Gates Foundation that includes funding grants. M-CM reports a relationship with French Dairy Interbranch Organization, Sodiaal-Candia and Danone that includes funding grants; relationship with Sodiaal-Candia that includes consulting or advisory; relationship with International Milk Genomics Consortium that includes speaking and lecture fees and travel reimbursement; relationship with Danone Nutricia Research and French Dairy Interbranch Organization that includes travel reimbursement. SS-M reports a relationship with Dutch Dairy Association and Danish Dairy Research Foundation that includes funding grants.
